# Mapping the locus for ocular melanosis in Cairn Terriers

**DOI:** 10.1111/vop.13291

**Published:** 2024-10-24

**Authors:** Paige A. Winkler, Ethan M. Dawson‐Baglien, Madeline C. Coffey, Patrick J. Venta, Kari J. Ekenstedt, Simon M. Petersen‐Jones

**Affiliations:** ^1^ Michigan State University Veterinary Medical Center East Lansing Michigan USA; ^2^ Department of Basic Medical Sciences, College of Veterinary Medicine Purdue University Indiana USA

**Keywords:** Cairn Terrier, dog, iris, ocular melanosis, pigmentary dispersion syndrome, secondary glaucoma

## Abstract

**Objective:**

To map the disease locus for familial ocular melanosis (OM) in the Cairn Terrier.

**Animals Studied:**

Cairn Terriers with OM and normal control dogs.

**Procedure:**

A genome‐wide association study (GWAS) was performed using 63 OM‐affected and 31 control Cairn Terriers, followed by haplotype analysis. A significantly associated single‐nucleotide polymorphism was genotyped in a larger group of OM‐affected and control Cairn Terriers. The coding and splice‐site regions of genes mapping within the confidence interval were sequenced.

**Results:**

A ~9.2 Mb region of chromosome 11 was significantly associated with OM. Haplotype analysis narrowed the region to 1.49 Mb. Genotyping of a SNP within the region showed 86% of OM‐affected dogs were homozygous or heterozygous for the risk allele, whereas 78% of unaffected dogs were homozygous for the nonrisk allele. Sequencing of the coding regions and splice sites of four genes (*c9orf72*, *IFNK*, the 5′ end of *MOB3B*, and the 3′ end of *LINGO2*) and of a microRNA (MIR876) did not detect any genetic variants unique to OM‐affected dogs.

**Conclusion:**

OM in Cairn Terriers maps to a 1.49 Mb region of chromosome 11. This accounts for 86% of OM cases in our DNA bank. A second locus may account for the OM phenotype in the remaining 14% of cases. Sequencing of coding regions and splice sites of positional candidate genes and a microRNA did not reveal any genetic variants unique to affected dogs. Further studies are required to elucidate the DNA variant causal for OM in Cairn Terriers and to understand the disease mechanism.

## 1 INTRODUCTION

Breed‐related ocular melanosis (OM) is a condition in dogs characterized by a proliferation of anterior uveal pigment‐laden cells, the majority of which are melanocytes.^1^, ^2^ In severely affected dogs, secondary glaucoma can result, leading to blindness and pain often necessitating enucleation. The condition is bilateral, and lesions are similar between the two eyes, although if glaucoma develops it does not typically do so simultaneously in both eyes. OM is most prevalent in Cairn Terriers, although a clinically similar condition is reported in other breeds including the Boxer and Labrador Retriever.^3^ The condition has been categorized into five clinical stages although it is really a continuum of changes that develop with expansion of the pigmented cells and pigment migration or release from the iris.^1^ Initial manifestation is a thickening of the iris root due to expansion with pigment‐laden cells, predominantly melanocytes.^2^ These melanocytes frequently migrate to other parts of the eye, such as the sclera, choroid/tapetum, optic nerve head, and optic nerve meninges.^1^ The melanocytes are plump and packed with melanosomes of variable size.^2^ Melanophages are often also identified on immunohistochemical examination of affected enucleated eyes. A prominent early feature is the progressive development of very darkly pigmented sclera/episcleral plaques which expand with disease progression. The plaques usually develop initially in the ventral sclera just posterior to the limbus and become larger in this region. With further progression of the condition secondary glaucoma develops because of occlusion of the aqueous drainage angles with the released pigmented material. The deposition of pigment in the drainage angle can be seen by gonioscopy well before glaucoma develops and is more pronounced ventrally.^1^ Some dogs have episodes of anterior uveitis associated with shedding of pigment from the face of the iris into the aqueous seen as pigmented anterior chamber cells and regions of reduced iris pigmentation. There is a range of ages of onset and variable rates of disease progression in OM‐affected Cairn Terriers. Some dogs develop secondary glaucoma at around 7 years of age, whereas other dogs may be examined in their teens and found to only have the early stages of the condition (Petersen‐Jones, unpublished observations).

OM in Cairn Terriers was first reported in 1984 in the proceedings of the ACVO conference with subsequent reports within the peer‐reviewed literature.^1^, ^2^, ^3^, ^4^, ^5^, ^6^ Initial candidate gene‐based attempts to map the causative locus for OM examined 11 genes known to have a role in melanocyte and melanosome development or known to be associated with other pigmentation‐related diseases; this included a pair of genes (*Gpnmb* and *Tyrp1*) which have known mouse genetic variants that, together, produce a mouse phenotype (DBA/2J) with similar histopathological changes to OM as seen in Cairn Terriers.^7^, ^8^ Examination of SNP and microsatellite markers near these genes showed that no single common allele was present in all OM‐affected Cairn Terriers tested for any of these 11 genes. Pedigree analysis suggested OM was an autosomal‐dominant trait^1^; therefore, the lack of a common shared allele at these 11 loci was taken as evidence to exclude the loci as harboring a genetic variant causative for OM.^7^

Here, we report the results of a genome‐wide association study (GWAS) of 94 Cairn Terriers (63 cases and 31 controls) allowing us to map the OM locus to a ~1.49 megabase (Mb) region of chromosome 11.

## 2 MATERIALS AND METHODS

### 2.1 Sample collection

All DNA used in this study was extracted from blood or buccal swab samples from Cairn Terriers collected with owner consent. Standard protocols were used for DNA isolation. Samples were collected by owners (cheek swabs), by referring veterinarians (blood or cheek swabs) or at Michigan State University (MSU, blood or cheek swabs). All procedures were in compliance with the Association for Research in Vision and Ophthalmology (ARVO) statement for the Use of Animals in Ophthalmic and Vision Research. Procedures were approved by the MSU Institutional Animal Care and Use Committee (AUF number 04‐05‐054‐00, 05‐08‐076‐00, 05‐11‐106‐00, 05/16‐089‐00).

### 2.2 Whole‐genome canine SNP array

A total of 94 Cairn Terriers (63 cases and 31 controls Table S1) were genotyped on the Illumina CanineHD Genotyping BeadChip.^9^ Initially, 48 samples were genotyped by GeneSeek (Lincoln, Nebraska) on the Illumina CanineHD BeadChip using 173 662 single‐nucleotide polymorphism markers (SNPs) (19 unaffected controls, 29 OM‐affected). An additional set of 48 samples (two dogs were repeated from the original data set, 12 unaffected controls and 34 OM‐affected) were genotyped at the University of Minnesota Genomics Core Facility (Minneapolis, MN) using a newer model of the Illumina BeadChip with 220 853 markers, including the original 173 662 markers on the previous BeadChip. Unaffected controls were selected from a pool of samples collected from dogs that were 7 years or older and were reported by a board‐certified veterinary ophthalmologist as not showing signs of OM.

### 2.3 Data analysis

Whole‐genome SNP data were lifted over from CanFam3 to the CanFam4/UU_Cfam_GSD_1.0 assembly and standard quality control measures were conducted in PLINK1.9.^10^ All positions reported herein correspond to CanFam4. SNPs with more than 10% missing genotypes, with a minor allele frequency of less than 0.05, or with an extreme deviation from Hardy–Weinberg equilibrium (0.000 001) were pruned. Individual samples that had more than 10% missing genotypes were also pruned. A GWAS was performed in GEMMA,^11^ with the remaining 113 721 genetic variants and 89 dogs (61 cases and 28 controls). A relationship matrix was used in GEMMA to correct for genetic relatedness stratification. Manhattan plots and Q–Q plots were generated in R Studio (R version 4.3.0).^12^ Haplotype analysis using SHAPEIT^13^ was used to delineate the haplotype of the affected animals (Table S2).

Heritability of OM was estimated using three different prediction programs. Concomitant with the GWAS calculation in GEMMA, the same relationship matrix was used to estimate the phenotypic variance explained by the 113 721 SNPs. Similarly, heritability was estimated in GCTA (version 1.93.2),^14^ which also calculates a kinship matrix; here, the restricted maximum likelihood model was used. Finally, heritability was estimated in the BLUPF90^15^, ^16^ family of programs, three of which were used here: RENUMF90, THRGIBBS1F90, and POSTGIBBSF90. A total of 1 000 000 iterations were run in THRGIBBS1F90, with a burn‐in of 200 000, and saving every 10 iterations.

Genes within the ~1.49 Mb region of interest identified by the haplotype analysis were analyzed in VarElect (ve.genecards.org) using the keywords “melanoma OR iris OR pigment OR melanosome OR melanocyte OR melanin” (Table S3).

### 2.4 PCR‐RFLP genotyping

The UCSC genome browser (https://genome.ucsc.edu/) CanFam4 reference sequence was used to retrieve sequence information near the top GWAS SNPs. The web tool NEBcutter3.0 was used to determine whether any SNPs led to the loss or gain of a restriction enzyme cut site (https://nc3.neb.com/NEBcutter/). The SNP ultimately used for genotyping was located at chr11:46653851 (*C* < A, rs22142771). Primers to amplify this genetic variant were designed using Primer3 (http://bioinfo.ut.ee/primer3‐0.4.0/; 5'‐AAGTGACTTGCAGTGAGTGG‐3′ and 5′‐TGCCTTTCTCTGCCTCCTC‐3′). See Appendix S1 for details of the developed SNP RFLP genotyping assay.

### 2.5 Screening positional candidate genes

Four positional candidate genes and one microRNA (*MOB3B*, *IFNK*, *c9orf72*, *LINGO2*, and MIR876, respectively) were screened for coding region variations in seven dogs (three unaffected Cairn Terriers, three affected Cairn Terriers homozygous for the risk allele and one control dog from a different breed). Primer synthesis (Table S5) and PCR product sequencing were conducted through Eurofins Genomics (Louisville, KY).

## 3 RESULTS

### 3.1 Genome‐wide SNP array

**FIGURE 1:**
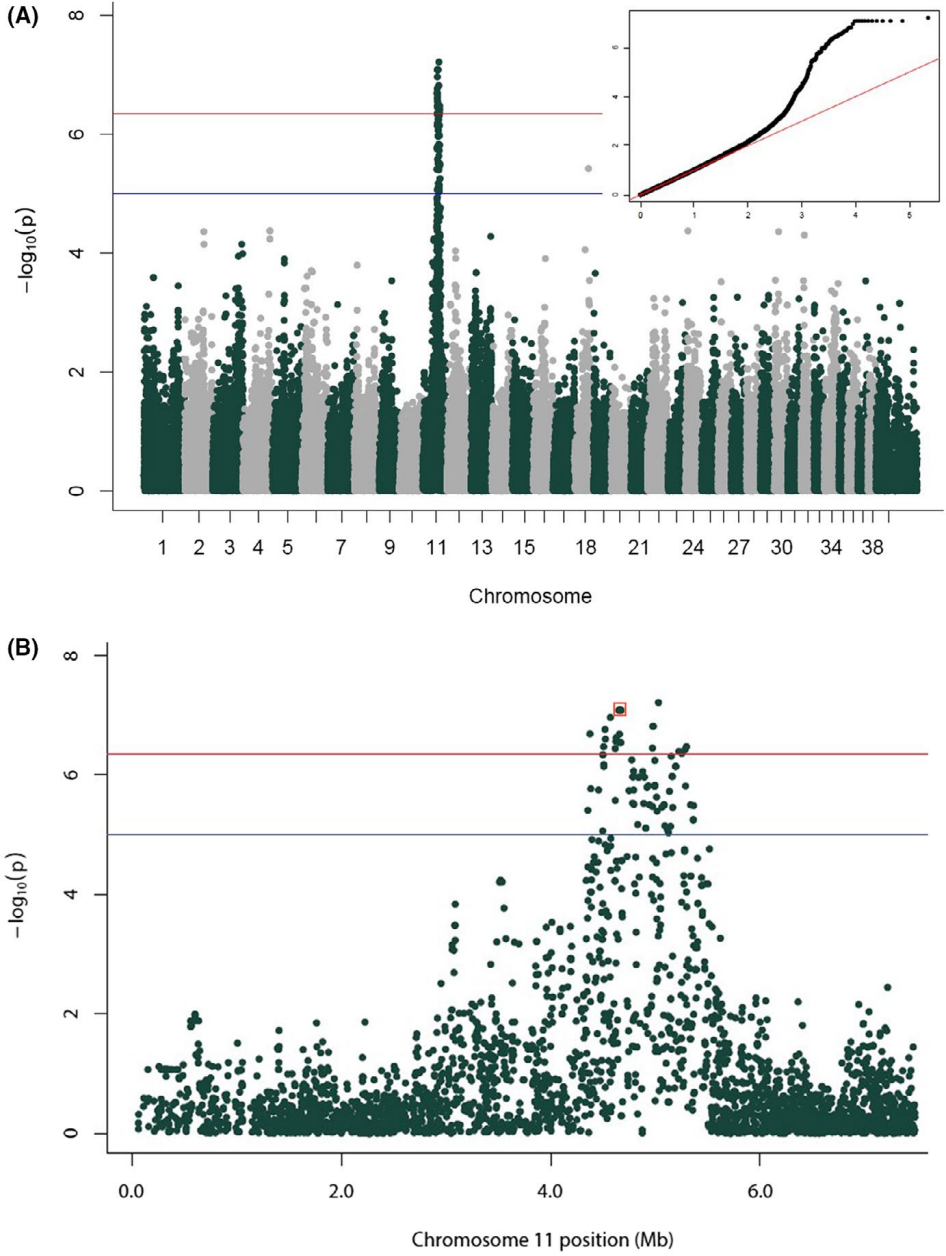
(A) Manhattan plot of the –log of the *p*‐values plotted against the genomic location for each of the 113 723 SNP markers genotyped in 88 dogs from the Illumina CanineHD BeadChip. The red line represents statistical significance (−log(4.4 × 10^−7^)). (B) Manhattan plot of chromosome 11 only. The linked SNP is boxed in red. The red line represents statistical significance (−log(4.4 × 10^−7^)).

A genome‐wide SNP array was conducted on 94 dogs, 63 affected, and 31 unaffected Cairn Terriers. The mean (±SD) age of the affected dogs at sampling was 11.27 ± 2.37 years; 24 were males and 39 females; 40 of the 63 affected dogs (63.5%) had developed glaucoma and the mean (±SD) age of the unaffected dogs at sampling was 11.93 ± 2.5 years; 13 males and 18 females (Table S1). Quality control pruning of SNPs resulted in 113 723 genetic variants for analysis. The filters removed 3636 SNPs due to low genotyping rate (>10% of samples not genotyped), 35 402 SNPs due to low minor allele frequency (MAF < 5%) and 2 SNPs were removed for deviations from Hardy–Weinberg equilibrium. Of the 94 dogs genotyped, 89 dogs passed the genotyping rate threshold (61 affected dogs and 28 unaffected dogs). The total genotyping rate for the 89 remaining dogs was >99.4%. Five dogs were filtered out for genotyping rates of less than 90% (Table S1). GEMMA used a relationship matrix to correct for genetic relatedness stratification (Figure 1A, inset Q‐Q plot) and GWAS demonstrated a significant region on chromosome 11 (Figure 1A,B). According to the Bonferroni correction for multiple testing, the genome‐wide significance threshold for a test involving 113 723 markers is *p* < 4.4 × 10^−7^. Thirty SNPs from the combined BeadChip data had significant *p*‐values, ranging from 6.13 × 10^−8^ to 4.39 × 10^−7^ (Table S4). These SNPs formed a block on chromosome 11, delineating a ~9.2 Mb region (chr11:43747423‐52 971 776) that was associated with the OM phenotype at statistically significant levels. No SNPs from other areas of the genome reached statistical significance (Figure 1).

Haplotype break points were identified in at least three affected dogs which allowed for further defining the region of interest. This analysis showed that the associated haplotype ranged from chr11:45865318 to 47356270 reducing the ~9.2 MB region to 1.49 MB (Table S2). The most significant SNP (chr11:50303636, *p*‐value 6.13 × 10^−8^) was outside of the haplotype break points and was not closely “clustered” with other neighboring significant SNPs. Heritablity calculations confirmed that OM is a genetic trait as previously published (Table 1).^1^

**TABLE 1:** SNP‐based heritability of ocular melanosis calculated in multiple programs.

Program	Heritability
GEMMA ± SE	0.749 ± 0.237
GCTA ± SE	0.758 ± 0.240
BLUPF90 ± PSD	0.901 ± 0.118

Abbreviations: PSD, posterior standard deviation; SE, standard error; SNP, single‐nucleotide polymorphism.

### 3.2 Population SNP genotyping

The 10 SNPs with the lowest *p*‐values within the 1.49 Mb region were assessed for RFLP testing. All 10 of these SNPs had the same *p*‐value of 8.24 × 10^−8^ (Table S4). Only one SNP (chr11:46653851 *C* > A, rs22142771) contained a restriction enzyme cut site and additional control cut site(s). This SNP was used to genotype all affected Cairn Terriers (163 dogs; 1–16 years; *M* = 59, *F* = 99, Unknown sex = 5) in our sample database. Additionally, Cairn Terriers reported as unaffected by a board‐certified veterinary ophthalmologist and at least 7 years or older were included as controls (95 dogs; 7–16 years; *M* = 47, *F* = 47, Unknown sex = 1) (Table 2). In total, 86% of the affected Cairn Terriers (140/163) were genotyped as homozygous or heterozygous for the linked marker, whereas 78% of the unaffected Cairn Terriers genotyped as homozygous wild type (74/95). A Fisher's exact test provides a significance of 6.9 × 10^−26^.

**TABLE 2:** Population‐wide SNP genotyping results.

OM status	Genotype	Number of dogs
Unaffected	A/A	1
	A/C	20
	C/C	74
Affected	AA	44
	A/C	96
	C/C	23

Abbreviation: SNP, single‐nucleotide polymorphism.

Examination of the transcripts annotated within the 1.49 Mb region on the Uppsala University Genes Track^17^ on the UCSC Genome Browser (https://genome.ucsc.edu/) shows four putative transcribed genes, some of which have multiple splice variants, and one microRNA (MIR876). These genes were assessed for keywords associated with the OM disease phenotype (see Methods) using VarElect (Table S3). None of the genes within this region achieved a high score with VarElect (which we defined as >10) and additional examination of the published literature did not immediately identify a functional candidate gene.

### 3.3 Screening of positional candidate genes

The four genes (*c9orf72*, *IFNK*, the 5′ end of *MOB3B*, and the 3′ end of *LINGO2*) and MIR876 which mapped to the 1.49 Mb region were screened for genetic variants in coding exons and adjacent intronic (splice) regions. The only noncoding exons that were not screened were exons with greater than 80% GC content or the extended 3′UTR. Genes and primers are shown in Table S5. The regions were sequenced in seven dogs (three affected Cairn Terriers, three older unaffected Cairn Terriers, and one dog from another breed). The affected Cairn Terriers used for this work were specifically selected to be homozygous for the disease‐associated genetic variant of the population SNP described above. No genetic variants unique to the affected dogs within the coding and splice sites of these four genes and MIR876 were identified on sequencing. Variations between canfam4 and OM‐affected dogs are listed in Table S6.

## 4 DISCUSSION

A GWAS of ocular melanosis‐affected Cairn Terriers identified a 9.2 Mb portion of canine chromosome 11 that is significantly associated with the phenotype. Haplotype analysis further narrowed the region to 1.49 Mb. Using a single SNP located in the most statistically significant region, genotyping of 163 OM‐affected dogs in our sample database showed that the disease‐associated genetic variant was present in one or two copies in 86% (140/163) of all dogs affected with OM. Previous pedigree analysis suggested that OM segregates as an autosomal dominant trait in the Cairn Terrier.^1^ It is significant that 23 of 163 (14%) affected dogs did not have the risk‐associated marker and were thus not associated with the major OM‐linked allele. This finding suggests genetic heterogeneity in the condition. It is possible that misdiagnosis, sample mix up, and/or phenocopies/genocopies could account for some of the samples purported to be from OM‐affected dogs that did not have the risk allele. However, many of the discordant affected dogs had advanced OM, making a misdiagnosis in those cases unlikely. It seems plausible that the selection for a “dark eye” as described in breed literature could have resulted in more than one molecular cause for the bilateral and symmetrical proliferation of uveal melanocytes that is the main feature of ocular melanosis and results in a “dark eye.” Genetic heterogeneity has been reported in several inherited canine conditions including neurological and retinal disease.^18^, ^19^, ^20^ Further studies are required to investigate a potential separate locus responsible for OM in the subset of affected Cairn Terriers that do not have the chromosome 11 risk allele. The narrowed 1.49 Mb region on chromosome 11 harbors four genes and one microRNA. The coding exons and flanking splice sites were all screened by Sanger sequencing in affected dogs for a potential disease‐causing genetic variant, with no success. The only noncoding exons that were not Sanger sequenced were those that could not be PCR‐amplified due to high GC content and some extended 3'UTR regions (Table S5). However, heritability calculations demonstrate OM to be a highly heritable condition (estimates ranging from 0.749 to 0.901), which supports the significance of the associated chromosome 11 region and may lend support to our genocopy hypothesis.

The lack of a coding or splice genetic variant in genes within the mapped OM region in affected dogs could be explained if the causal genetic variant was in noncoding DNA that played an important role such as a regulatory region, microRNA, or long noncoding RNA; alternatively, a structural genetic variant could be present. Further studies, including whole‐genome sequencing, are underway to investigate these possibilities.

In summary, a GWAS study allowed us to identify a 1.49 Mb region of chromosome 11 significantly associated with OM in about 86% of OM‐affected Cairn Terriers. This information may allow a linked‐marker DNA test to be developed to help reduce the incidence of OM within the Cairn Terrier population.

## AUTHOR CONTRIBUTIONS

**Paige A. Winkler:** Formal analysis; investigation; methodology; writing – original draft; writing – review and editing. **Ethan M. Dawson‐Baglien:** Conceptualization; formal analysis; investigation; methodology; writing – original draft; writing – review and editing. **Madeline C. Coffey:** Formal analysis; methodology; writing – review and editing. **Kari J. Ekenstedt:** Formal analysis; investigation; resources; writing – review and editing. **Patrick J. Venta:** Formal analysis; writing – review and editing. **Simon M. Petersen‐Jones:** Conceptualization; funding acquisition; investigation; supervision; writing – original draft; writing – review and editing.

## FUNDING INFORMATION

Funding for this project was provided by the Foundation of the Cairn Terrier Club of America, Morris Animal Foundation (D17CA‐073), the AKC Canine Health Foundation (03094‐MOU), the Myers‐Dunlap Endowment For Canine Health, and MSU Endowed Research Fund.

## CONFLICT OF INTEREST STATEMENT

The authors declare no conflict of interest with respect to the research, authorship, and/or publication of this article.

## ETHICS STATEMENT

All procedures were in compliance with the Association for Research in Vision and Ophthalmology (ARVO) statement for the Use of Animals in Ophthalmic and Vision Research. Procedures were approved by the MSU Institutional Animal Care and Use Committee (AUF number 04‐05‐054‐00, 05‐08‐076‐00, 05‐11‐106‐00, and 05/16‐089‐00).

## Supporting information


Appendix S1

Tables S1–S6

## Data Availability

Data that support the findings of this study are available upon request.
