# Supplementary material for: Mapping the locus for ocular melanosis in Cairn Terriers
Source: Vet Ophthalmol. 2024 Oct 24;29(1):e13291. doi: 10.1111/vop.13291 (PMC12748030; doi:10.1111/vop.13291)
Supplement: Supplementary file 1 — Appendix S1 [file VOP-29-0-s002.docx]

Supplemental Methods

A restriction fragment length polymorphism (RFLP) assay was designed to assess the shared affected haplotype in a larger number of Cairn Terriers. It was important for the authors to choose an inexpensive enzyme that also had a control cut site(s) within the product. The control cut site(s) ensures that the enzyme digest was complete in all samples. Primers are highlighted in blue and the restriction enzyme cut sites (MluCI, AATT) are highlighted in yellow. The SNP site is demarcated by brackets with the nucleotide associated with the disease phenotype highlighted in purple. There are two internal cut site controls, creating two bands at 44 and 60 bps. The diagnostic bands are in purple text. The digested samples can be easily viewed on a 2% agarose gel (45 minutes at 170 volts). L1 – 100 bp ladder, L2 – Affected – heterozygous, L3 – Affected – homozygous for diseases-associated variant, L4 – Unaffected – homozygous wild-type

chr11:46653851, rs22142771

AAGTGACTTGCAGTGAGTGGCCTCTGGAGTAGCTGTTGAAAGATCTGGAAAAACTTAATTGTTAGAACAACACACTGTGTTGCTCTTTTTTTCAGTGTAAAATTCCAGGGCGACTATACCTTTCAA[C/A]TTGGTCAATGAACTTTGAACTGAAGAATCTCATATGGAGGAGTTCAGAGATTCCACTCTTAATCAGATTAAAAGAGCTAACTCAATGAGGAGGCAGAGAAAGGCA


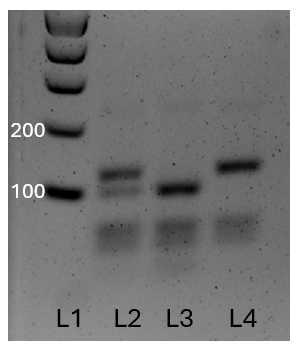


Forward Primer - AAGTGACTTGCAGTGAGTGG

Reverse Primer - TGCCTTTCTCTGCCTCCTC

Product Size 232 bps

Enzyme MLUCI – cut site AATT

Wild type cuts: 44, 60, 128

Affected cuts: 25, 44, 60, 103
